# Insights and future directions for the application of perinatal derivatives in eye diseases: A critical review of preclinical and clinical studies

**DOI:** 10.3389/fbioe.2022.969927

**Published:** 2022-11-08

**Authors:** María Norte-Muñoz, Maria Filomena Botelho, Andreina Schoeberlein, João Chaves, Joaquim Neto Murta, Peter Ponsaerts, Marta Agudo-Barriuso, Esmeralda Costa

**Affiliations:** ^1^ Experimental Ophthalmology Group, IMIB-Arrixaca, University of Murcia, Murcia, Spain; ^2^ Institute of Biophysics and Institute for Clinical and Biomedical Research (iCBR), Area of Environment Genetics and Oncobiology (CIMAGO), Faculty of Medicine, University of Coimbra, Coimbra, Portugal; ^3^ Center for Innovative Biomedicine and Biotechnology (CIBB), University of Coimbra, Coimbra, Portugal; ^4^ Clinical and Academic Centre of Coimbra (CACC), Coimbra, Portugal; ^5^ Department of Obstetrics and Feto-maternal Medicine, Inselspital, Bern University Hospital, University of Bern, Bern, Switzerland; ^6^ Department for BioMedical Research (DBMR), University of Bern, Bern, Switzerland; ^7^ Ophthalmology Department, Centro Hospitalar e Universitário de Coimbra, Coimbra, Portugal; ^8^ Ophtalmology Universitary Clinic and Institute for Clinical and Biomedical Research (iCBR), Faculty of Medicine, University of Coimbra, Coimbra, Portugal; ^9^ Laboratory of Experimental Hematology, Vaccine and Infectious Disease Institute (Vaxinfectio), University of Antwerp, Antwerp, Belgium

**Keywords:** perinatal derivates, ophthalmology, preclinical models, clinical trials and database search, mesenchymal stromal cells

## Abstract

Perinatal derivatives (PnD) are gaining interest as a source for cell-based therapies. Since the eye is easily accessible to local administration, eye diseases may be excellent candidates to evaluate novel therapeutic approaches. With this work, we performed a systematic review of published preclinical and clinical studies addressing PnD in the treatment of ocular diseases. We have set two specific objectives: (i) to investigate the current level of standardization in applied technical procedures in preclinical studies and (ii) to assess clinical efficacy in clinical trials. Hereto, we selected studies that applied amniotic membrane (hAM) and mesenchymal stromal cells derived from amniotic membrane (hAMSC), placenta (hPMSC), umbilical cord (hUC-MSC) and Wharton’s Jelly (hUC-WJ-MSC), excluding those where cells were not transplanted individually, following a systematic PubMed search for preclinical studies and consultation of clinical studies on https://clinicaltrials.gov and https://www.clinicaltrialsregister.eu/. Our bibliographic search retrieved 26 pre-clinical studies and 27 clinical trials. There was a considerable overlap regarding targeted ocular structures. Another common feature is the marked tendency towards (i) locally administered treatments and (ii) the PnD type. In the cornea/ocular surface, hAM was preferred and usually applied directly covering the ocular surface. For neuroretinal disorders, intra-ocular injection of umbilical or placental-derived cells was preferred. In general, basic research reported favourable outcomes. However, due to lack of standardization between different studies, until now there is no clear consensus regarding the fate of administered PnD or their mode of action. This might be accountable for the low index of clinical translation. Regarding clinical trials, only a minority provided results and a considerable proportion is in “unknown status”. Nevertheless, from the limited clinical evidence available, hAM proved beneficial in the symptomatic relief of bullous keratopathy, treating dry eye disease and preventing glaucoma drainage device tube exposure. Regarding neuroretinal diseases, application of Wharton’s Jelly MSC seems to become a promising future approach. In conclusion, PnD-based therapies seem to be beneficial in the treatment of several ocular diseases. However, much is yet to be done both in the pre-clinical and in the clinical setting before they can be included in the daily ophthalmic practice.

## 1 Introduction

Cell therapy encompasses a wide range of treatment strategies that use cells as therapeutic agents. Although cellular therapies have multiple beneficial properties, the rationale supporting the application of cellular medicines varies depending on the source of cells, the target organ or disease. Focusing on research using stem cells, based on their developmental status they can be classified into (i) embryonic stem cells (ESC) and (ii) adult stem cells (ASC). ESCs are extracted from the inner cell mass of blastocysts. They actively divide and applied as such are tumorigenic *in vivo*, which, together with their implicit ethical problems, means that they are of little benefit to patients in an undifferentiated stage. ASC encompass a wide variety of (stem) cell types that can be obtained postnatal from neonate to adulthood, but also from extra-embryonic perinatal tissues. Several examples of well-known ASC are those derived from bone marrow, dental pulp or adipose tissue (AT). They are widely investigated in novel regenerative medicine strategies because they can be isolated from the patient with little discomfort, and there are no ethical concerns. However, the efficacy of ASC therapy, apart from bone marrow transplantation for hematological malignancies, varies depending on their source, the age of the donors and, importantly, their health (Torre and Flores, 2020). On the other hand, perinatal derivatives (PnD) are prepared from fetal discarded annexes (i.e., amniotic membrane, placenta, umbilical cord). These tissues not only have been less exposed to infections and diseases, come from “young tissue” and are not burdened with ethical concerns, but also could be used as biological structure or scaffold (Abbaspanah et al., 2018).

Three decades ago, the first therapeutic mesenchymal stromal/stem cells (MSC) were isolated from bone-marrow (BM) (Moll et al., 2019). However, throughout the years, MSC originating in other locations have gained increasing interest, with PnD representing 27% and AT up to 22% of the chosen MSCs in registered clinical trials between 2008 and 2018 (Moll et al., 2019). But in contrast to BM, whose safety has been well established, AT and PnD have been associated with severe adverse effects when administered intra-vascularly, some of them fatal. These adverse reactions involve pro-thrombotic and pro-inflammatory mechanisms that were demonstrated to be linked with the presence of high levels of procoagulant Tissue Factor (TF) in those cells (Caplan et al., 2019; Moll et al., 2019; Moll et al., 2022). In addition, intra-vascular injection activates the host immune system, which on the one hand seems to convey the MSC immunomodulatory function, but on the other hand, promotes the sequestration and inactivation of MSC, thereby decreasing their potential therapeutic effect (Moll et al., 2019).

There is an increasing body of evidence from pre-clinical and clinical studies showing that those deleterious effects can be minimized with several strategies, such as modifying MSC manufacture, improving MSC characterization, establishing optimal concentrations and administering simultaneous anticoagulant therapy (Moll et al., 2019; Caplan et al., 2019; Moll et al., 2022). In this context, local administration *via* direct injection in the target organ/tissue, when possible, presents some advantages. In theory, it can eliminate the need for hemocompatibility and minimize the risk of life-threatening complications [thrombosis and severe systemic inflammatory reaction—Instant Blood-Mediated Inflammatory Reaction (IBMIR)]. Over the years, there has been a tendency among clinical trials to move from intra-vascular delivery to locally administered treatments, with the latter being the choice in about half of the clinical trials currently performed (Moll et al., 2019).

The eye is, *per se*, considered a sanctuary, protected from the bloodstream by hemato-ocular barriers, rendering him a target difficult to reach endovenously, similarly to central nervous system (Caplan et al., 2019). On the other hand, it is easily accessible, either topically or by intra-ocular administration (intra-vitreal, intra-cameral, subconjunctival, subtenon injection). These surgical procedures are widely and routinely performed worldwide, in an outpatient basis and are well tolerated, with little discomfort. Local administration to the eye easily bypasses the ocular barriers, probably increasing cell delivery efficiency and lowering the incidence of complications (Caplan et al., 2019), which makes the eye a suitable candidate for PnD-based therapies.

With this work, we aimed to perform a systematic review of published preclinical and clinical studies involving therapeutic approaches of PnD in the field of ocular diseases. We have set forward two specific objectives: (i) to investigate the current level of standardization in applied technical procedures, regarding cell therapeutic interventions in preclinical studies, and (ii) to assess how clinical efficacy of PnD was evaluated in view of current and future clinical relevance

This work was performed in the framework of The International Network for Translating Research on Perinatal Derivatives into Therapeutic Approaches (SPRINT, CA17116), funded by COST (European Cooperation in Science and Technology).

## 2 Materials and methods

We performed a review of published preclinical and clinical studies selecting those that used perinatal derivatives (PnD). We included amniotic membrane and mesenchymal stromal cells derived from amniotic membrane (hAMSC), placenta (hPMSC), umbilical cord (hUC-MSC) and Wharton’s jelly (hUC-WJ-MSC), excluding those where cells were not transplanted individually or were not used in combination with other derivatives such as conditioned medium.

For preclinical studies, the SPRINT consortium performed a systematic search of the PubMed database (Linares-Espinós et al., 2018) using a Boolean search string including perinatal derivatives such as tissues, membranes, cells, and secretome derived from them, but excluding cord blood and hematopoietic products, as well as non-original publications. The search string was supplemented with terms covering preclinical animal or *in vivo* models, and publications from 2004 to the present were collected in a searchable database as described in detail elsewhere (https://doi.org/10.5281/zenodo.6334077). The filter “*ophthalmology OR eye OR vision OR retina OR cornea*” was then used to select preclinical studies in the ophthalmology field, that were manually curated to exclude adult MSC.

The search for Clinical Trials (CTs) using PnD in the treatment of ocular conditions was carried out in the https://clinicaltrials.gov and https://www.clinicaltrialsregister.eu/databases, using the following terms: “*Ophthalmopathy OR ocular OR vision OR eye OR ophthalmic OR Eye Disease OR Eye OR Ocular OR optic OR retina AND mesenchymal”*. Next, the retrieved CTs (*n* = 41) were manually curated to exclude those testing adult MSCs (*n* = 14) (Supplementary Figure S1). In addition, CTs that used hAM as a carrier for other cell types, such as in limbal cell transplant, were excluded from this search, since their primary goal was not the use of AM. We also excluded CTs that had been withdrawn. Data were analyzed and plotted using GraphPad Prism 9 (GraphPad, San Diego, CA, United States).

## 3 Results

Our bibliographic search, as outlined in Materials and Methods section, retrieved 26 pre-clinical studies and 27 randomized clinical trials (CTs), that are listed in Tables 1, 2.

**TABLE 1:** Papers selected for pre-clinical studies and PnD analysis. (PMID: PubMed ID; PnD: Perinatal Derivates; RCS: Royal College Surgeons; hUC-MSC: human Umbilical Cord Mesenchymal Stromal Cells; hAM: human Amniotic Membrane; hAMSC: human Amniotic Membrane Mesenchymal Stromal Cells; hPMSC: human Placenta Mesenchymal Stromal Cells; hUC-WJ-MSC: human Umbilical Cord Wharton’s Jelly Mesenchymal Stromal Cells).

PMID	Animal	Injury	Tissue	PnD	Administration	Immunosuppression	Size of experimental groups	Outcome
31523119 Call et al. (2019)	Col5a1^Δst/Δst^ and Col5a1^f/f^ mice	Keratectomy	Cornea	hUC-MSC	Single subconjunctival injection	No	Not mentioned	Reduction of corneal opacity at 7 days in treated Col5a1^f/f^ with no improvement at 14 days. Significant reduction in Col5a1^Δst/Δst^ mice at 7 and 14 days
31727008 Zhou et al. (2019)	C57BL/6J mice	Fungal keratitis	Cornea	hUC-MSC	Repeted subconjunctival injection	No	*n* = 6 for each group	Collagen destruction was restored by uMSCs treatment with an AOD 69,97 ± 7.09 at 14 days post-injury, vehicle group (28.98 ± 3.32)
16249483 Heiligenhaus et al. (2005)	BALB/c mice	Viral keratitis	Cornea	hAM	Sutured to cornea	No	*n* = 12 for each group	Severity of corneal keratitis is significant reduced after 2 days. (1.2± 0.8 vs. no transplanted 3.1 ± 1.1)
17637463 Bauer et al. (2007)	BALB/c mice	Viral keratitis	Cornea	hAM	Sutured to cornea	No	*n* = 12 for each group	Severity of corneal keratitis is significantly reduced after 2 days of AMT (1.2 ± 0.8 vs. no transplanted 3.1 ± 1.1)
19255156 Bauer et al. (2009)	BALB/c mice	Viral keratitis	Cornea	hAM	Sutured to cornea	No	Not specifically mentioned in transplants but from *in vitro* assays n = 8	AM reduces infiltering cells but not well detailed
18172088 Barequet et al. (2008)	Wistar rats	Bacterial keratitis	Cornea	hAM	Sutured to cornea	No	*n* = 16 saline group *n* = 15 antibiotics group *n* = 16 antibiotics-AM	One week after transplantation, significant reduction of opacity, and neovascularization in AM group than antibiotics (*p* value = 0.007) and saline group (*p* = 0.014)
24695478 Zeng et al. (2014)	New Zealand white rabbits	Alkali burns	Cornea	hAMSC	Single subconjunctival injection	No	vehicle *n* = 10, for all transplanted groups *n* = 12	At 7 days, control (PBS) exhibited significant higher opacity score (3.9) than transplanted ones (hAMSC 3.15, AM graft 3.20 and combination 2.45)
28704317 Subasi et al. (2017)	New Zealand white rabbits	Alkali burns	Cornea	hAM	Sutured to cornea	No	*n* = 8 for each group	Less neovascularization was observed in the collagen group than in the AM group. No differences in opacity
21431286 Reza et al. (2011)	New Zealand white rabbits	Keratectomy	Cornea	hAM	Sutured to cornea	No	*n* = 4 for each group	AM exhibited more transparent up to 10 weeks after injury with no neovascularzation or irregularities
29929442 Yamashita et al. (2018)	Japan white rabbits	Keratectomy	Cornea	hUC-MSC	Sutured to cornea over a collagen sheet	No	*n* = 6 for each group	At 8 days after transplantation, red cells can be detected in transplant group and there is no edema
21310014 Guo et al. (2011)	New Zealand white rabbits	Keratectomy	Cornea	hAM	Sutured to cornea	No	*n* = 11 for each group	At 7 days the diameter of lesion decreased significantly in AM group
30260581 Navas et al. (2018)	New Zealand white rabbits	Alkali burns	Cornea	hAMSC	Single injection in anterior chamber	No	*n* = 6 for each group	At 12 days after injury, hAMSC group presents a signifcant inhibition (0.5 ± 0.05 vs. NaOH 4.11 ± 0.26) of neovascularitation and the lowest corneal opacity (1.24 ± 0.06 vs. NaOH 3.21 ± 0.34)
31399042 Park et al. (2019)	BALB/c mice	Graves’ Ophthalmopathy induced by immunization with hTSHR	Optic nerve surrounding tissues	hPMSC	Single intraorbital injection	No	*n* = 12 in saline + *n* = 14 steroids + *n* = 14 hPMSC	At 4 weeks hPMSC decrease % of orbital volume like steroid treatment but also attenuate pro inflammatory cytokine production
34051850 Park et al. (2021)	BALB/c mice	Graves’ Ophthalmopathy induced by immunization with hTSHR	Optic nerve surrounding tissues	hPMSC	Single intraorbital injection	No	*n* = 6 for each group	1 week after transplantation, hPMSCs^PRL1^ and hPMSCs found to reduce the thickness of the optic nerve but steroid did not have the same effect. Also, WAT (white adipose tissue) area immunodeteted by perilipin was significantly reduced in hPMSC group
25734497 Xie et al. (2015)	Sprague Dawley rats	Optic nerve transection	Optic nerve	hAMSC	Single intranerve injection	No	n = 18 for sham and n = 21 for the rest of groups	28 days after transplantation, GAP-43 integral optical density value in control group was lower than transplantation group (*p* < 0.0001)
28857477 Park et al. (2018)	Sprague Dawley rats	Optic nerve crush	Optic nerve	hPMSC	Single intranerve injection	No	*n* = 5 for each group	2 weeks after injection axon survival ratio was higher in the hPMSC group
32524519 Kwon et al. (2020)	Sprague Dawley rats	Optic nerve crush	Optic nerve	hPMSC	Single subtenon injection	No	*n* = 4 for sham and *n* = 6 for transplanted groups	4 weeks after transplantation, expression of GAP43 was significantly increased in all treated groups
30389962 Millán-Rivero et al. (2018)	Sprague Dawley rats	Optic nerve crush	Retina and optic nerve	hUC-WJ-MSC	Single intravitreal injection	No	*n* = 4 for each group	At 7 days, hUC-WJ-MSCs protected retinal ganglion cells from death after axotomy
29210653 Wang et al. (2017)	RCS rats	Photoreceptor degeneration	Retina	hUC-MSC	Single subretinal injection	No	*n* = 6 for each group	8 weeks after transplantation there are GFP positive cells in both groups. Between 1 and 8 weeks after transplantation, hUC-MSCs demonstrated significant protective effect in b waves of ERG.
26107378 Leow et al. (2015)	RCS rats	Photoreceptor degeneration	Retina	hUC-WJ-MSC	Single subretinal injection	Yes Dexamethasone + Cyclosporine A	*n* = 8 for each group	At 2 weeks, cells remained in subretinal localization. No significant differences between groups in waves amplitude of ERG at 15 and 30 days. However, outer nuclear layer (ONL) thickness at 70 days was higher in transplanted group (*p* < 0.001)
17053209 Lund et al. (2007)	RCS rats	Photoreceptor degeneration	Retina	hUC-MSC and hPMSC	Single intrascleral injection	Yes Dexamethasone + Cyclosporine A	*n* = 23 for hUC-MSC group *n* = 8 for hPMSC group	At 1 month, hUC-MSCs rescued amplitude of ERG waves and not hPMSCs
21629576 Scalinci et al. (2011)	Lewis rats	Diabetic ophthalmopathy induced by streptozotocin	Retina	hPMSC	Single intravitreal injection	No	*n* = 3 for sham and *n* = 12 for transplanted groups	At 21 days, hPMSC group presented less hypofluorescence areas with means less isquemic zones than sham group. Only qualitative
32210708 Yu et al. (2020)	Sprague Dawley rats	Diabetic ophthalmopathy induced by streptozotocin	Retina	hUC-MSC	Single tail-vein injection	No	*n* = 15 for each group	After 1 month, hUC-MSCs reduced retinal microvascular permeability (*p* < 0.05)
31737079 Ji et al. (2019)	Sprague Dawley rats	Ocular hypertension (OHT)	Retina	hUC-MSC	Single intravitreal injection	No	*n* = 12 for each group	After 14 days, in OHT animals retinal thickness decreased significantly (89.09 ± 3.04 μm) while hUC-MSCs significantly increased that value (105.4 ± 3.37)
26065854 Kim et al. (2016)	C57BL/6J mice	Oxygen-induced retinopathy (OIR) by hyperoxic room	Retina	hAMSC	Single intraperitoneal injection	No	*n* = 18 for each group	After 5 days, hAMSCs were found surrounded vasculature in retina but not differentiated into endothelium or pericytes
22550516 Kiilgaard et al. (2012)	Domestic pigs	Choroidal neovascularitation induction (CNV) by removing RPE	Retina	hAM	Single subretinal injection	No	*n* = 9 control and *n* = 15 transplanted group	At 42 days, AM was covered by RPE cell in a monolayer

**TABLE 2:** Clinical trials selected for the present review. (hAM: human Amniotic Membrane; hAMSC: human Amniotic Membrane Mesenchymal Stromal Cells; hUC-MSC: human Umbilical Cord Mesenchymal Stromal Cells; hUC-WJ-MSC: human Umbilical Cord Wharton’s Jelly Mesenchymal Stromal Cells).

Type of tissues	Type of cell used	Route of administration	Ocular therapeutic target	Disease treated	RCT References	Status	Published results
hAM	hAM as a tissue	over tissue	Cornea	Bullous keratopathy	https://ClinicalTrials.gov/show/NCT01926535	Completed	yes
hAM	AM Extract Eye Drop	topical	Cornea	Epithelial defects	https://ClinicalTrials.gov/show/NCT02746848	Completed	No
hAM	hAM as a tissue	over tissue	Cornea	Corneal perforation	https://ClinicalTrials.gov/show/NCT03500796	Completed	No
hAM	hAM as a tissue	over tissue	Cornea/Dry Eye	Dry Eye	https://ClinicalTrials.gov/show/NCT04553432	Recruiting	No
hAM	AM Extract Eye Drop	topical	Cornea	Limbal insufficiency	https://ClinicalTrials.gov/show/NCT02649621	Completed	No
hAM	hAM as a tissue	over tissue	Cornea	Limbal insufficiency	https://ClinicalTrials.gov/show/NCT02102776	Unknown	No
hAM	hAM as a tissue	over tissue	Cornea	Limbal insufficiency	https://ClinicalTrials.gov/show/NCT02015000	Unknown	Submitted
hAM	hAM as a tissue	over tissue	Cornea	Limbal insufficiency	https://ClinicalTrials.gov/show/NCT01319721	Completed	Yes
hAM	hAM as a tissue	over tissue	Cornea	Corneal ulcer	https://ClinicalTrials.gov/show/NCT01765244	Completed	no
hAM	hAM as a tissue	over tissue	Scleral	Scleral thinning	https://clinicaltrials.gov/ct2/show/study/NCT00801073	Unknown	Yes
hAM	hAM as a tissue	over tissue	Cornea	Bullous keratopathy	https://clinicaltrials.gov/ct2/show/study/NCT00659308	Completed	yes
hAM	hAM as a tissue	over tissue	Cornea	Limbal insufficiency	https://clinicaltrials.gov/ct2/show/study/NCT00457223	Completed	No
hAM	hAM as a tissue	over tissue	Cornea	Limbal insufficiency	https://clinicaltrials.gov/ct2/show/study/NCT00802620	Unknown	No
hAM	hAM as a tissue	over tissue	Cornea	Corneal ulcer	https://clinicaltrials.gov/ct2/show/study/NCT00915759	Unknown	Yes
hAM	hAM as a tissue	over tissue	Cornea	Corneal ulcer/melting	https://clinicaltrials.gov/ct2/show/study/NCT02168790	Completed	Yes
hAM	hAM as a tissue	over tissue	Cornea	Corneal ulcer	https://clinicaltrials.gov/ct2/show/study/NCT00238862	Completed	No
hAM	hAM as a tissue	over tissue	Sclera/conjunctiva	Shunt tube exposure	https://clinicaltrials.gov/ct2/show/study/NCT01551550	Completed	Yes
hAM	hAM Extract Eye Drop	topical	Cornea/Dry Eye	Dry Eye	https://www.clinicaltrialsregister.eu/ctr-search/trial/2011-006287-50/ES	Prematurely ended	Yes
hAM	hAM conditioned media	topical	Cornea/Dry Eye	Dry Eye	https://clinicaltrials.gov/ct2/show/study/NCT02369861	Completed	No
hUC	UC MSC	not stated (systemic administration?)	Optic nerve	Neuromyelitis Optica/Multiple sclerosis	https://ClinicalTrials.gov/show/NCT01364246	Unkonown	No
hUC	UC MSC	subconjunctival injection	Cornea	Ocular Corneal Burn	https://ClinicalTrials.gov/show/NCT03237442	Unknown	No
hUC	UC MSC and UC MSC-Exo	intravitreal injection	Retina	Macular Hole	https://ClinicalTrials.gov/show/NCT03437759	Active, not recruiting	No
hUC	UC MSC-Exo	topical	Cornea/Dry Eye	Dry eye/GVHD	https://ClinicalTrials.gov/show/NCT04213248	Recruiting	No
hUC	WJ MSC	subtenon injection	Retina	Retinitis pigmentosa	https://ClinicalTrials.gov/show/NCT04224207	Completed	yes
hUC	UC MSC and UC MSC conditioned media	peribulbar injection	Retina	Retinitis pigmentosa	https://ClinicalTrials.gov/show/NCT04315025	Completed	No
hUC	UC MSC	subtenon injection and suprachoroidal injection	Retina	Retinitis pigmentosa	https://ClinicalTrials.gov/show/NCT04763369	Recruiting	No
hUC	WJ MSC	subtenon space	Optic nerve	Toxic optic neuropathy	https://ClinicalTrials.gov/show/NCT04877067	Completed	Yes

**FIGURE 1:**
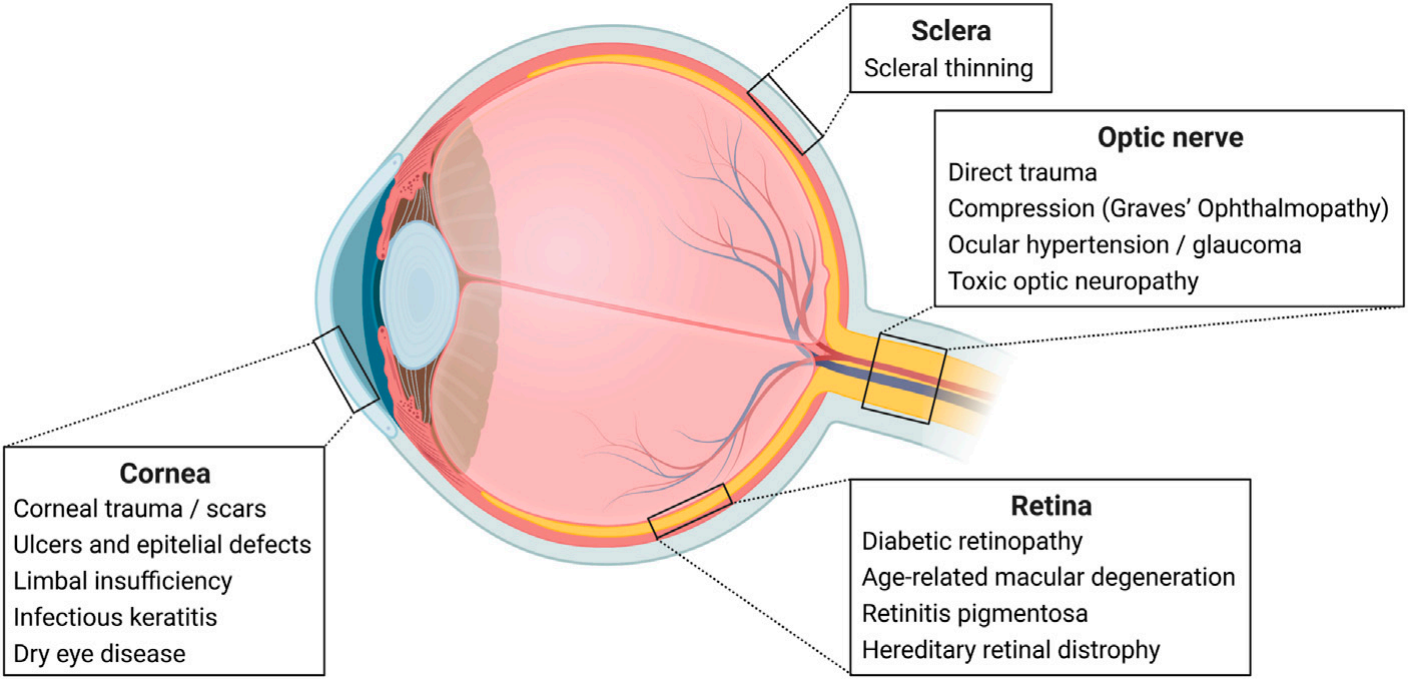
Ocular Anatomy and Diseases. Examples of eye diseases per ocular structure. Created with BioRender.com.

Both pre-clinical studies and CTs addressed diseases of the cornea/ocular surface and the retina/optic nerve (Figure 1). No other ocular structure was object of study.

### 3.1 Preclinical studies: Animal models

**FIGURE 2:**
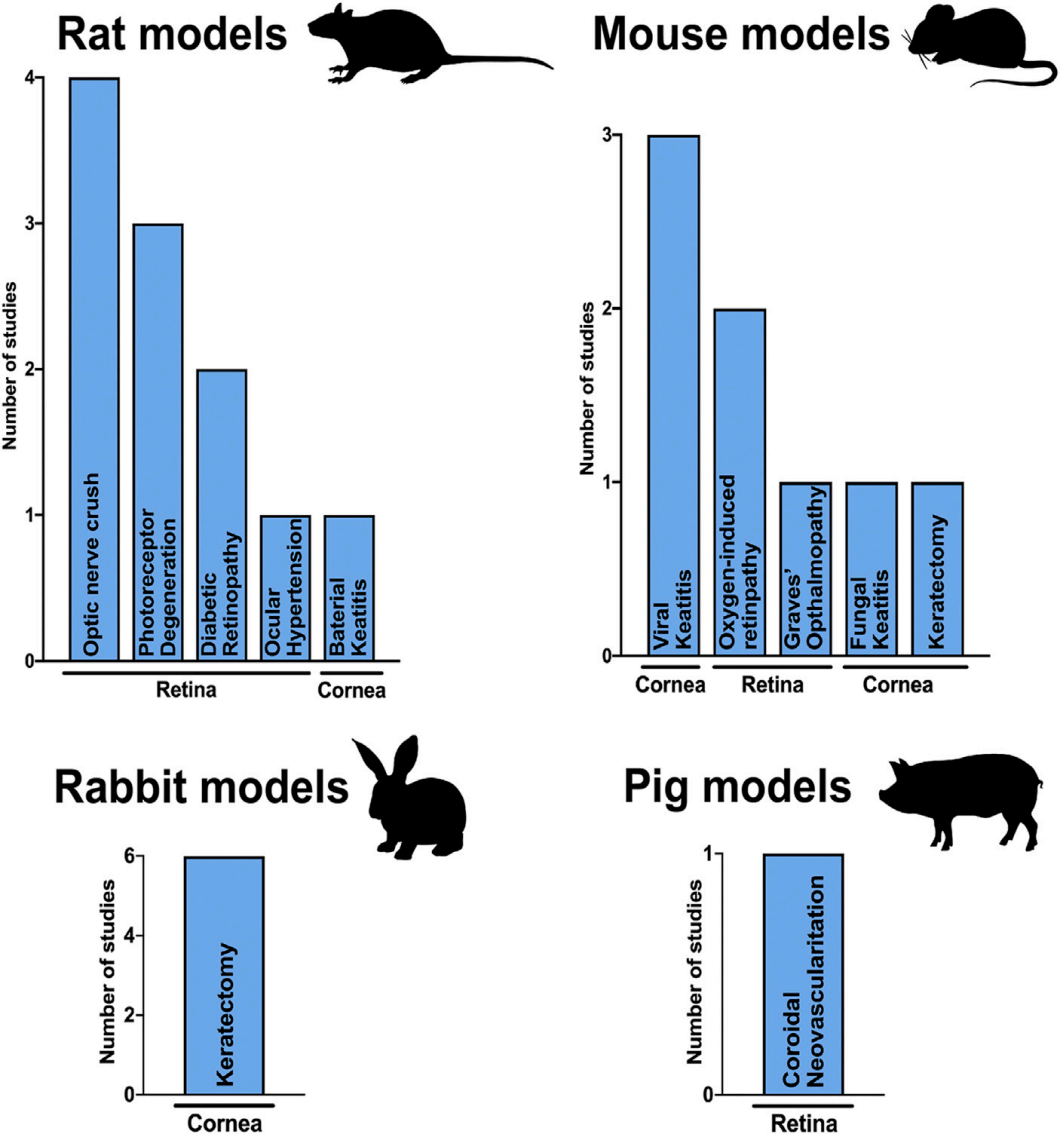
Animal models. Column graphs showing the species used to test PnDs in ophthalmology and the number of articles published for every injury model in the cornea and retina.

Most of the reports studied the effects of PnD in rats (*n* = 11), mice (*n* = 8) and rabbits (*n* = 6). Only one of them used higher mammals, namely domestic pigs, to assess the effect of amniotic membrane in retinal neovascularization (Kiilgaard et al., 2012). The used species and applied disease models are summarized in Table 1 and Figure 2.

Rabbits are exclusively used as keratectomy model (Figure 2), where corneal ablation is performed by placing a NaOH soaked disk over the cornea (Guo et al., 2011; Reza et al., 2011; Zeng et al., 2014; Subasi et al., 2017; Navas et al., 2018; Yamashita et al., 2018).

Rodents are the most common model to study either keratitis using bacterial (Barequet et al., 2008), fungal (Zhou et al., 2019) or viral infection (Heiligenhaus et al., 2005; Bauer et al., 2007; Barequet et al., 2008) and retinal injuries or diseases such as photoreceptor degeneration (Lund et al., 2007; Leow et al., 2015; Wang et al., 2017), optic nerve trauma (Xie et al., 2015; Millán-Rivero et al., 2018; Park et al., 2018; Kwon et al., 2020), hypoxia (Kim et al., 2016), ocular hypertension (Ji et al., 2019), Grave’s ophthalmopathy (Park et al., 2019, 2021) and diabetic retinopathy (Scalinci et al., 2011; Yu et al., 2020).

All selected papers analyzed a large enough sampling size (12 papers had a “number of animals” higher than 10) to perform consistent statistical analyses. Furthermore, according to the ethical committees’ recommendations, most of the studies explicitly mentioned the age or weight of animals, although neither explored how transplantation effects differ among interindividual variables.

### 3.2 Preclinical studies: Type of cells and route of administration

**FIGURE 3:**
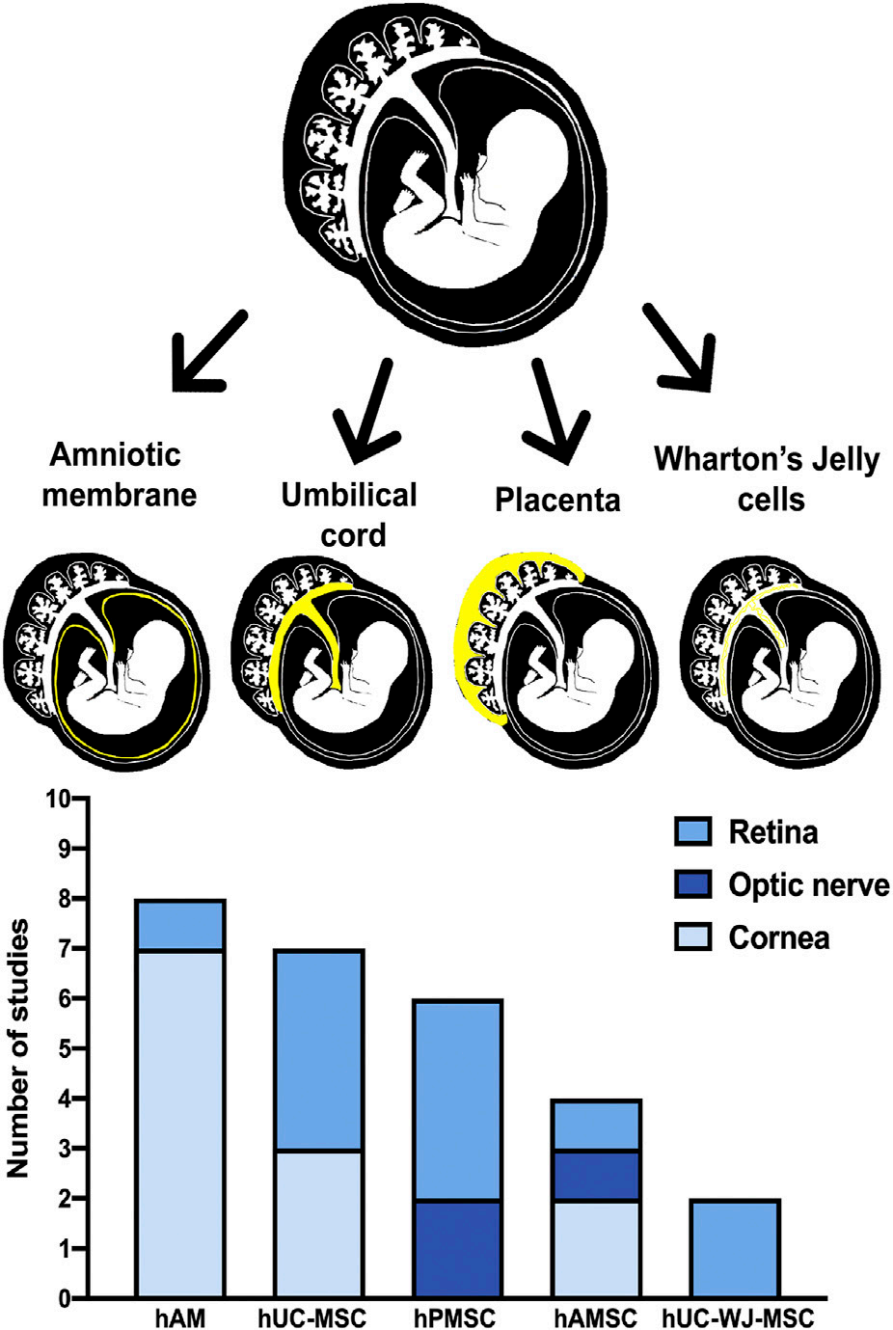
Types of PnD. Top, a schematic picture of different origins of fetal tissue for each PnD selected in this review. Below, grouped column graph showing the number of publications using each PnD type in retina, optic nerve, and cornea. (hAM: human Amniotic Membrane; hUC-MSC: human Umbilical Cord Mesenchymal Stromal Cells; hPMSC: human Placenta Mesenchymal Stroma Cells; hAMSC: human Amniotic Membrane Mesenchymal Stromal Cells; hUC-WJ-MSC: human Umbilical Cord Wharton’s Jelly Mesenchymal Stromal Cells).

As we expected for their translational potential to the clinic, all the selected studies used human PnDs (Figure 3); thus, in these works, PnDs were xenotransplanted. Surprisingly, among all 26 studies, only two applied an immunosuppressive regimen (Lund et al., 2007; Leow et al., 2015).

Regarding corneal diseases, we identified 12 studies using PnD: five aiming to treat corneal infections, four evaluating corneal epithelialization in keratectomy models and three evaluating its effect in alkali burns. The majority (*n* = 8) was treated with hAM as an over tissue, three with subconjunctival injections of hUC-MSC or hAMSC and one with an intra-cameral injection of hAMSC (Heiligenhaus et al., 2005; Bauer et al., 2007, 2009; Barequet et al., 2008; Guo et al., 2011; Reza et al., 2011; Kiilgaard et al., 2012; Subasi et al., 2017).

There were 14 studies focusing on retinal and optic nerve disorders. In contrast to corneal pathologies, umbilical cord derived cells were preferred (*n* = 6), followed by hPMSC (*n* = 5) and hAMSC (*n* = 2).

In retinal diseases (*n* = 8), subretinal injections (*n* = 3) and intra-vitreal injections (*n* = 2) were the most frequently performed procedures. Of notice, there were two studies where cells were systemically administered, either *via* the tail or injected in intra-peritoneal space. In none of those two studies systemic immunosuppression was administered. (Lund et al., 2007; Wang et al., 2017; Yamashita et al., 2018; Call et al., 2019; Ji et al., 2019; Zhou et al., 2019; Yu et al., 2020) (Lund et al., 2007; Scalinci et al., 2011; Park et al., 2018, 2019, 2021; Kwon et al., 2020).

In optic nerve disorders (*n* = 6), cells were injected directly in the nerve (*n* = 2), in the orbit (*n* = 2), in the subtenon space (*n* = 1) and in the vitreous (*n* = 1).

### 3.3 Pre-clinical studies: Cell fate and outcome reports

A major strength of preclinical animal models is that they allow to determine the cell fate of transplanted cells. However, only ten studies identified the graft *post-mortem*, using diverse techniques that include immunofluorescence (Millán-Rivero et al., 2018), membrane staining with lipophilic fluorophores (Scalinci et al., 2011; Yamashita et al., 2018; Kwon et al., 2020) or nuclei stains (Xie et al., 2015; Navas et al., 2018). Of notice, Kim et al., 2016 proved that intraperitoneally injected hAMSCs can migrate into the retina, resulting in supressed neovascularization in the retina. The rest of studies that identified cells agreed on transplanted cells remaining in the local of injection (Millán-Rivero et al., 2018; Kwon et al., 2020), and their effects are locally potentiated but detectable all over retina.

**FIGURE 4:**
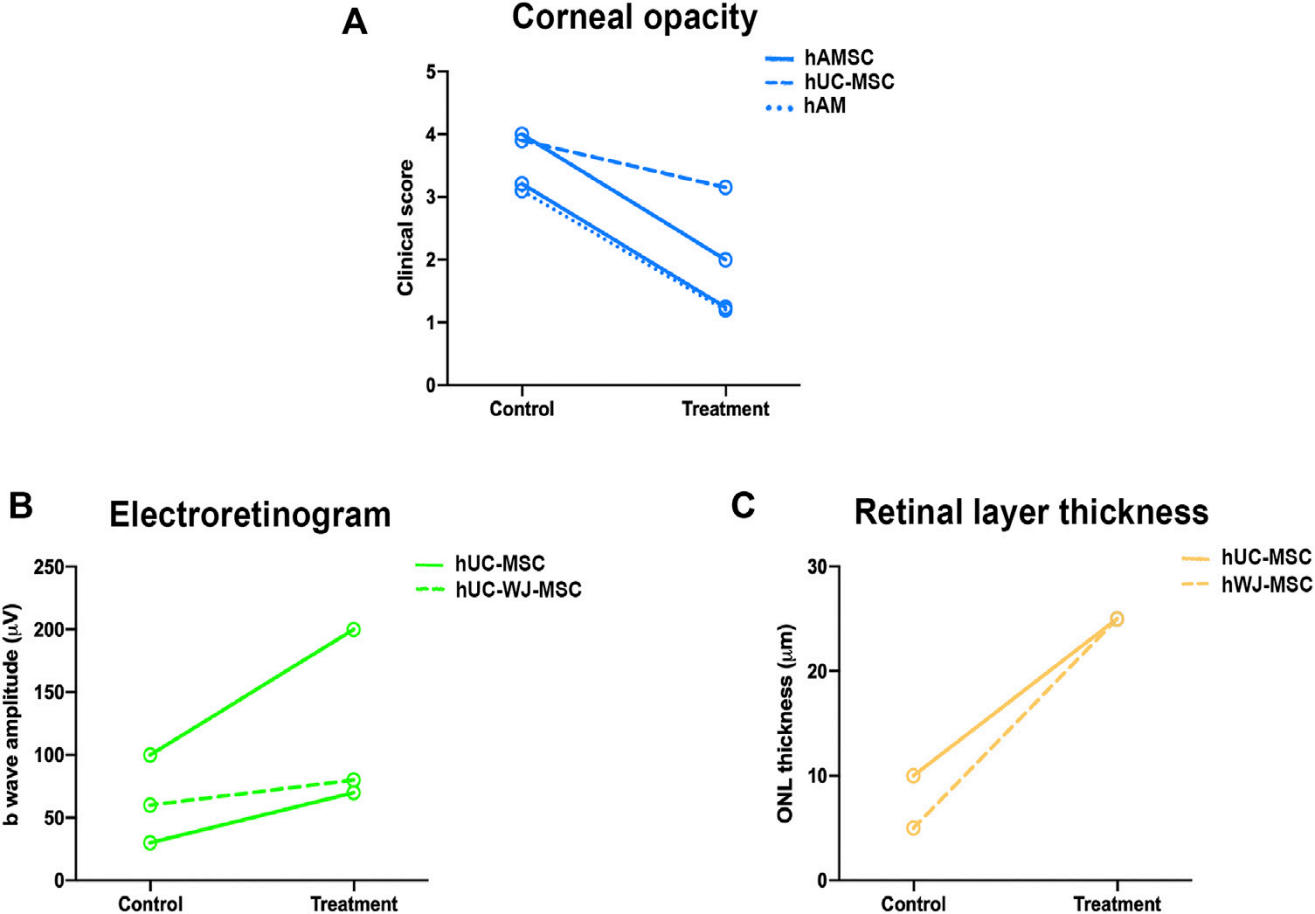
Preclinical scores to measure PnD therapeutic success. Graphs representing the functional or anatomical changes after PnD treatment between control and treated groups. Clinical score measuring corneal opacity in keratitis corneal model 15 days after the transplant **(A)**, b wave amplitude (µV) recorded from rats with retinal dystrophy measured 30 days after injection **(B)** and measurement of the thickness of outer nuclear layer (ONL) (µm) in the same model but measured at 60 days after injection **(C)**. (hAM: human Amniotic Membrane; hUC-MSC: human Umbilical Cord Mesenchymal Stromal Cells; hAMSC: human Amniotic Membrane Mesenchymal Stromal Cells; hUC-WJ-MSC: human Umbilical Cord Wharton’s Jelly Mesenchymal Stromal Cells).

Regarding the route of administration, most of the studies report beneficial effects of PnD when applied locally. The two studies that investigated systemic administration also report beneficial effects, with no severe adverse reactions (Kim et al., 2016; Yu et al., 2020). More concerning is the absence of consensus regarding the optimal time points for analyses, so that positive effects can be reported as early as 2 days post-administration (Heiligenhaus et al., 2005; Bauer et al., 2007) or 2 months (Reza et al., 2011; Wang et al., 2017) after transplantation. Furthermore, the approach for measuring the beneficial effect of PnD transplants also lacks consensus. As shown in Figure 4, we selected those studies that assessed functional or anatomical outcomes in the same animal model and matched time points: for cornea, the clinical score for improvement was the corneal opacity after transplant (Heiligenhaus et al., 2005; Zeng et al., 2014; Navas et al., 2018; Yamashita et al., 2018) and for the retina, recordings of the b-wave in electroretinograms that measure post photoreceptors synapsis (Lund et al., 2007; Leow et al., 2015; Wang et al., 2017)**.** In the studies graphed in Figure 4, PnD displayed a beneficial impact in decreasing corneal opacity, increasing the b-wave amplitude and ONL thickness, but results differed depending on PnD tissue origin. However, no clear statement can be made due to the low number of studies.

### 3.4 PnD in clinical trials

**FIGURE 5:**
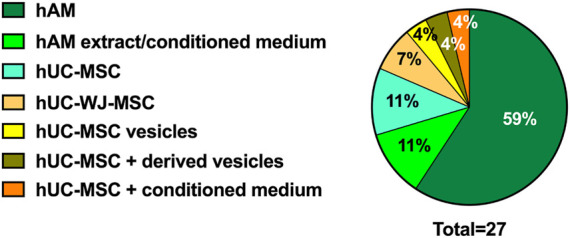
Randomized Clinical Trials (RCT) and PnD used. Pie graph representing the type of PnD used in RCTs.

Our curated search for Clinical Trials (CT) retrieved 27 registries, that are summarized in Table 2. The vast majority addresses the use of human amniotic membrane, either as a tissue (*n* = 16) or as an extract/conditioned medium eye drop (*n* = 3). The remaining trials evaluate the use of umbilical cord mesenchymal stromal cells (hUC-MSC) (*n* = 3), Wharton’s Jelly MSC (hUC-WJ-MSC) (*n* = 2), hUC-MSC-derived extracellular vesicles (*n* = 1), hUC-MSC plus hUC-MSC-derived extracellular vesicles (*n* = 1) and hUC-MSC plus hUC-MSC-conditioned media (*n* = 1) (Figure 5).

**FIGURE 6:**
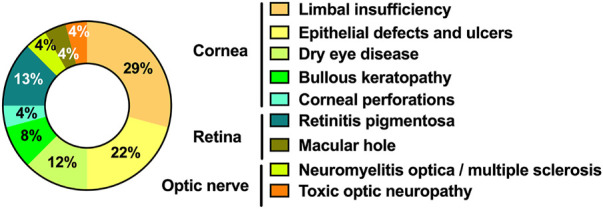
Ophtalmic diseases object of RCT grouped by ocular target. Circular graph representing the distribution of ocular diseases that were object of RCT.

The majority of CTs have used the cornea as a therapeutic target (*n* = 19) and are designed to treat limbal insufficiency (*n* = 7), epithelial defects and ulcers (*n* = 5), bullous keratopathy (*n* = 2), dry eye disease (*n* = 3), and corneal perforations (*n* = 1). The usefulness of hAM in treating scleral/conjunctival thinning is the object of two CTs (Figure 6).

**FIGURE 7:**
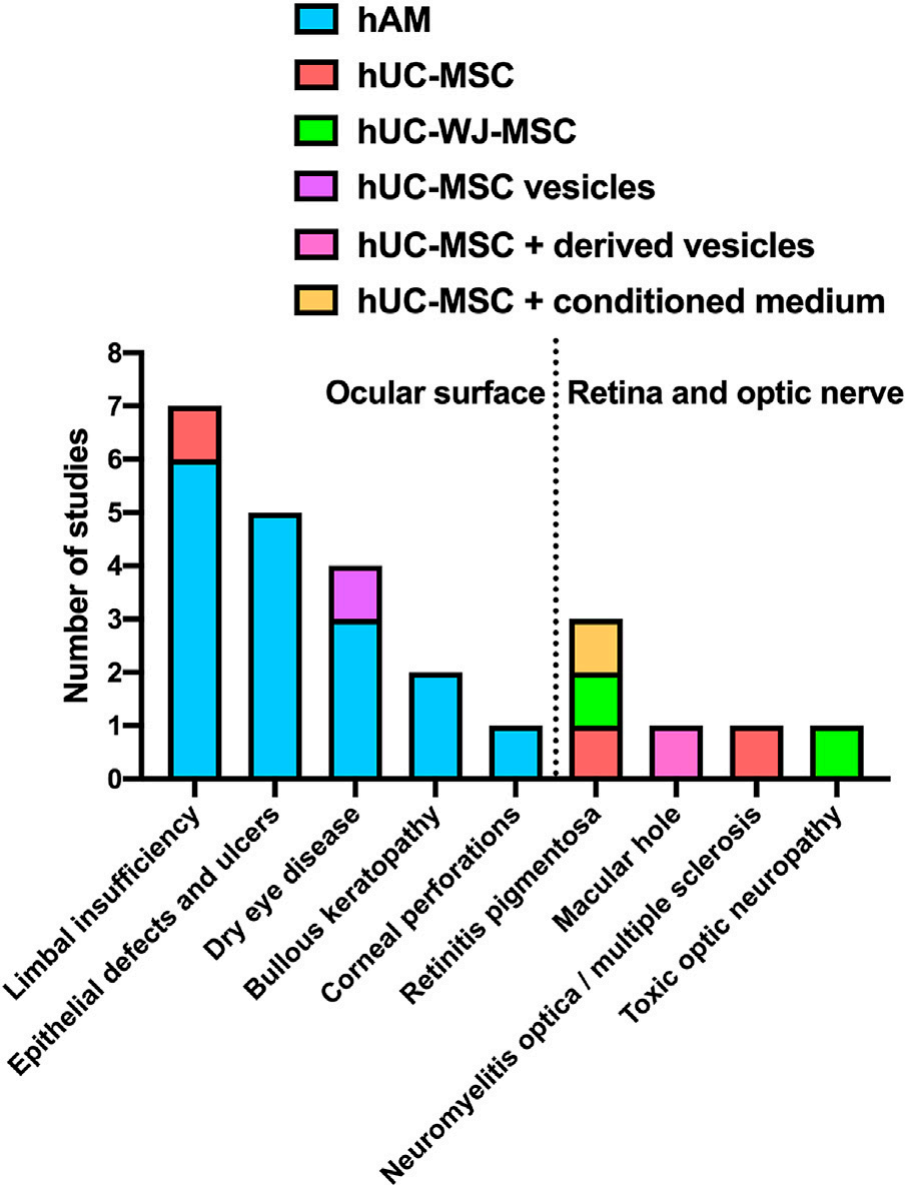
Type of PnD used in every disease. Column graph representing the type of PnD used in each pathology; the dashed line separates pathologies associated with the ocular surface on the left and the retina or optic nerve on the right.

The remaining six CT are designed to treat retinal diseases (*n* = 4) and optic neuropathies (*n* = 2): retinitis pigmentosa (*n* = 3), macular hole (*n* = 1), neuromyelitis optica/multiple sclerosis (n = 1) and toxic optic neuropathy (*n* = 1) (Figure 6). Of notice, all these six RCT are designed to evaluate the therapeutic value of umbilical cord and Wharton’s Jelly-derived products, rather than hAM, which is not a choice in treating these diseases (Figure 7).

In fact, it becomes apparent from Figure 7 that hAM is the PnD of choice to treat ocular surface conditions (the columns to the left of the dashed line), while retinal and optic nerve diseases are predominantly treated with hUC and hUC-WJ derivatives. The route of administration is inherent to that choice: while ocular surface conditions are treated with hAM applied to the surface or with PnD eyedrops, the retina and the optic nerve are not that easily accessible and therefore require PnD to be delivered through subconjunctival, subtenon, periocular or intravitreal injection (Table 2).

**FIGURE 8:**
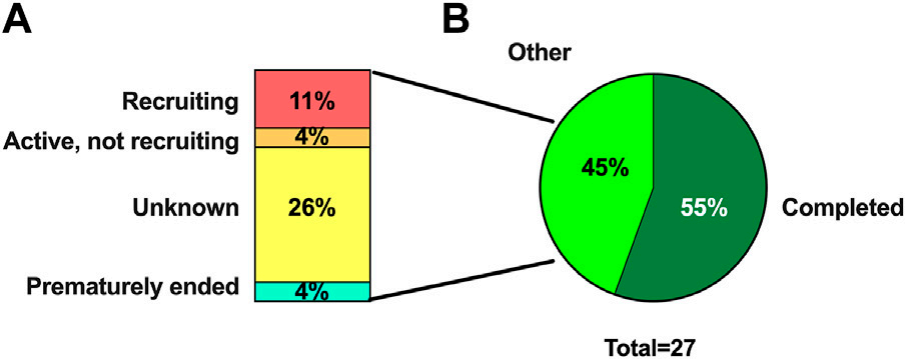
Recruiting status of RCT. Column graph **(A)** representing percentage of RCT not completed and circular graph **(B)** with percentage of RCT completed and not completed of twenty-seven RCT included.

Fifteen of those 27 RCT are currently completed, three are still recruiting, one is active but not recruiting, and seven have an unknown recruiting status (Figure 8). Surprisingly, only ten RCT have published results.

Available results are compiled in Table 3 and detailed below, by pathology.

**TABLE 3:** Clinical trials with published results.

References	Title/Purpose/Description	Condition	n° of eyes	Parameters evaluated
NCT01926535	Amniotic Membrane Graft in Symptomathic Bullous Keratopathy	Bullous keratopathy	AMT 10 CL 10	Eye pain, visual acuity; bullae, corneal epithelial defects, corneal neovascularization and complications (6 months)
	Amniotic Membrane Transplantation (AMT) vs. contact lens (CL)			
NCT00659308	Amniotic Membrane and Anterior Stromal Puncture to the Treatment of Symptomatic Bullous Keratopathy	Bullous keratopathy	AMT 20 ASP 20	Regular epithelium (6 months)
	Compare AMT and anterior stromal puncture (ASP) in the management of pain in patients with symptomatic bullous keratopathy			
NCT01319721	Recurrent Pterygium Surgery Using Mitomycin C With Limbal Conjunctival or Amniotic Membrane	Pterygium	47 (CLAU) + 42 (AMT)	Recurrence at 1Year
	Randomized, interventional, comparative: limbal-conjunctival autograft (CLAU) vs. AMT in recurrent pterygia			Healing Time of Corneal Defect
				Postoperative Conjunctival Inflammation
NCT01551550	Shunt Tube Exposure Prevention Study (STEPS)	Prevention of tube exposure	AMT 41 Peric 40	Tube exposure (2 Years)
	Randomized, interventional, comparative: AMT vs. pericardial graft covering glaucoma drainage device tube			
NCT00801073	Comparison Amongst Scleral, Corneal and Amniotic Membrane Grafts to Restore Scleral Thinning	Scleral thinning	Total 26	Increase in scleral thickness at 6 months
	Compare the use of multilayer amniotic membrane transplantation (AMT) with lamellar corneal transplantation (LCT) and lamellar scleral transplantation (LST) for the treatment of scleral thinning after pterygium surgery			
NCT00915759	Sutureless Cryopreserved Amniotic Membrane Graft (ProKera) and Wound Healing After Photorefractive Keratectomy (PRK)	Corneal ulcer	40 patients AMT 40 CL 40	Corneal Re-epithelialization measured as number of days to complete re-epithelialization
	Compare sutureless AMT and bandage contact lens in aiding corneal re-epithelialization after PRK (both eyes of 40 patiens enrolled) Non randomized			
NCT02168790	Safety Study of a Sutureless Amniotic Membran Transplantation to Treat Ocular Surface Disorders (Expanded Access) (AmnioClip)	Corneal ulcer/erosion; Corneal scarring	8 eyes (7 patients)	Tolerance and complications
	Seven day-wearing period of AmnioClip to prove safety, wearability and fit of AmnioClip as a prerequisite to obtain a regulatory approval			
2011-006287-50	Phase III comparative clinical trial to evaluate the efficacy of amniotic membrane extract for the treatment of severe dry eye disease, in comparison with autologous serum eyedrops	Dry Eye	12 patients (6 AM. extract + 6 AS)	Schirmer Test, Fluorescein Clearance test, TBUT, Impression cytology: HLA-DR, MUC1; subjective improvement
	Amniotic membrane (AM) extract vs. autologous serum (AS)			
NCT04224207	Management of Retinitis Pigmentosa by Mesenchymal Stem Cells by Wharton’s Jelly Derived Mesenchymal Stem Cells (WJ-MSC)	Retinitis pigmentosa	34 eyes (32 patients)	outer retinal thickness, mean horizontal ellipsoid zone, BCVA, fundus perimetry deviation index, full-field flicker ERG parameters
	prospective, sequential, open-label phase-3 clinical study			
NCT04877067	Therapy of Toxic Optic Neuropathy *via* Combination of Stem Cells With Electromagnetic Stimulation (Magnovision)	Toxic Optic Neuropathy	36 eyes (18 patients)	best corrected visual acuity, fundus perimetry deviation index, ganglion cell complex thickness, visual evoked potential

#### 3.4.1 Bullous keratopathy

Two RCT evaluate the use of transplanted hAM in the management of BK (NCT01926535; NCT00659308). Both seem to favour hAM: hAM outperforms therapeutic contact lenses in pain relief up to 6 months and is more effective in achieving a regular epithelium at 6 months, compared to anterior stromal puncture.

#### 3.4.2 Pterygium

One randomized, interventional, and comparative study on recurrent pterygium surgery compared conjunctival-limbal autograft (CLAU) with hAM and concluded the superiority of CLAU in all parameters evaluated (healing of epithelial defect, post-operative conjunctival inflammation, and recurrence rate at 1 year) (NCT01319721).

#### 3.4.3 Scleral thinning

A randomized, interventional, comparative study compared hAM with pericardial graft covering to prevent glaucoma drainage device tube exposure and found that hAM was more effective at the 2-year time point (NCT01551550).

Another RCT compared the use of multilayer amniotic membrane transplantation (AMT) with lamellar corneal transplantation (LCT) and lamellar scleral transplantation (LST) for the treatment of scleral thinning after pterygium surgery (NCT00801073). It found that AMT performed significantly worse than LCT or LST. A high rate of reabsorption was noted with AMT, which was the least effective of the three therapeutic options and should not be used for this condition.

#### 3.4.4 Corneal ulcer

Two RCTs evaluated the role of sutureless hAM in the treatment of corneal ulcers. One was designed to prove its safety and wearability as a prerequisite to obtaining regulatory approval. Eight eyes of seven patients were enrolled and showed good tolerance with no complications (NCT02168790).

The largest RCT compared hAM and bandage contact lens (BCL) in aiding corneal re-epithelialization after photorefractive keratectomy in 40 patients. One eye of each patient was treated with hAM, and the other eye with BCL. Published results show shorter healing times for BCL, but no statistical analysis was performed, so clear conclusions cannot be drawn (NCT00915759).

#### 3.4.5 Dry eye disease

One phase III comparative clinical trial evaluated the efficacy of amniotic membrane extract for treating severe dry eye disease compared with autologous serum eyedrops (2011-006287-50). Among the group of 12 patients enrolled, no statistical differences were noted between the two treatments. Since autologous serum eyedrops are a world-wide accepted treatment option for dry eye disease, this RCT proved the non-inferiority of hAM extract.

#### 3.4.6 Neuroretinal diseases

There are two RCTs with the focus on neuroretinal disease that have published results, both using hUC-WJ-MSC.

One prospective, sequential, open-label phase III clinical study enrolled 34 eyes of 32 patients with retinitis pigmentosa (RP) (NCT04224207). In this RCT, subtenon transplantation of WJ-MSCs was effective and safe in the treatment of RP during the first year.

Similarly, the beneficial effect of hUC-WJ-MSC combined with electromagnetic stimulation in toxic optic neuropathy was evidenced in another RCT involving 36 eyes of 18 patients (NCT04877067).

## 4 Discussion

Our thorough review of pre-clinical studies and clinical trials has shown a considerable overlap with regard to the ocular structures being targeted. The cornea, the retina and the optic nerve are the main targets of PnD therapeutic interventions. Another common feature is the marked tendency towards locally administered treatments, as opposed to systemic delivery. This is in agreement with the current world-wide trends in mesenchymal stromal/stem cell clinical applications (Moll et al., 2019).

Another similarity between pre-clinical and clinical studies is the preferred cell type used: in the cornea and ocular surface, hAM is the PnD of choice, while retinal and optic nerve diseases are predominantly treated with umbilical cord derived and placenta mesenchymal stromal cells. This also implicitly conditions the route of administration, ocular surface conditions being treated with hAM applied as a tissue or with PnD eyedrops, while the retina and the optic nerve are treated with injected cells.

The existence of clinical trials register platforms is a scientific, ethical and moral responsibility (https://www.who.int/clinical-trials-registry-platform/network/trial-registration). Databases for clinical trials registration are a valuable source of information. However, we noticed that the follow-up is frequently missing and results are not published; it is very common to find clinical trials in “unknown status”. Having a follow-up from clinical failure studies could also help to re-drive pre-clinical and clinical works to better targets.

The cornea is a specialized, transparent tissue placed in the anterior part of the eye, that allows the passage of light to the retina, the light sensory tissue (Figure 1). Thus, corneal integrity and transparency are essential for a clear vision. Once damaged, the cornea’s ability to heal relies on stem cells located around its periphery, the so-called limbal stem cells. Not unfrequently, this healing process leads to scar formation and transparency loss, especially in extensive lesions and long-standing disease. PnD-based therapies have immunoregulatory properties that may be useful in modulating wound healing (Bukowiecki et al., 2017). Both pre-clinical studies and CTs have addressed this subject, and demonstrated that PnDs are useful in corneal re-epithelialization in keratectomy animal models. In the clinical setting, hAM was proven useful in the symptomatic relief of bullous keratopathy and was well tolerated and safe in treating corneal ulcers and epithelial defects; however, it did not seem to outperform bandage contact lenses—only one CT with published results was performed, and it seemed to favour contact lens wear compared to hAM, but did not present statistical analysis.

Limbal insufficiency is an ocular surface disease involving the cornea that has attracted the attention of basic and clinical researchers. It is a devastating condition often leading to blindness, that is mostly caused by chemical injuries and typically affects young males, that are more prone to that kind of accidents (Deng et al., 2019). In limbal insufficiency, the cornea becomes opaque and neovascularized, which erodes ocular immune privilege (Bukowiecki et al., 2017) and therefore turns corneal grafts into very high-risk procedures due to the increased risk of rejection. The potential beneficial effect of PnD was evaluated in animal alkali burn models, with favourable results. Regarding clinical trials, there were seven registered studies. Of those, only one had their results published, and it evaluated pterygium, a localized form of limbal insufficiency that does not entail the severity of chemical burns. In pterygia surgery, hAM presented poor results, but the comparative treatment was conjunctival-limbal autograft, whose superiority is well known since it contains limbal stem cells.

There are other diseases of the ocular surface where the treatment with biological products has proven benefits. That is the case of dry eye disease, where the treatment with blood derived products is beneficial, due to the presence of growth factors, among other molecules, and is part of the current treatment guidelines (Jones et al., 2017). The discovery of other biological options that might be even more effective and wider available could be advantageous. In fact, hAM extract can be an option in treating dry eye disease, since a CT proved its non-inferiority compared to autologous serum eyedrops, a gold-standard treatment for this condition. hAM also proved helpful in preventing glaucoma drainage device tube exposure. On the contrary, a CT has shown that in the case of scleral thinning, the use of hAM should be discouraged since there are better alternatives (corneal or scleral grafts).

The optic nerve and retina are projections of the brain and therefore part of the central nervous system. They are highly specialized tissues where neuronal death can occur as a result of varied diseases: age-related macular degeneration (AMD), retinitis pigmentosa (RP), diabetic retinopathy (DR), trauma, ocular hypertension/glaucoma, etc. Regardless of their primary cause (mutations, aging, systemic disorder), these diseases share common underlying mechanisms, such as oxidative stress, excitotoxicity, inflammation, or cytokine imbalance (Holan et al., 2021). The current treatment for these diseases is varied, depending on the disease itself, but of limited success in visual recovery: it encompasses antiangiogenic intravitreal injections (for AMD and DR), laser applied to the retina and metabolic control (in DR), lowering intra-ocular pressure (in glaucoma), etc. There is currently no treatment available for end-stage retinal/optic nerve diseases, where the discovery of nerve cell regeneration therapies remains the holy grail. Of notice, two CT addressing neuroretinal diseases showed very promising results on the use of Wharton’s Jelly MSC, bringing hope to otherwise irreversible diseases. However, one must say that the current belief is that these cells do not differentiate into neurons, rather they facilitate neurorestorative mechanisms (Caplan et al., 2019).

When clinical trials and pre-clinical studies are compared, it becomes evident that research does not easily translate to the clinic, despite reported beneficial effects. That is especially noticeable in neuroretinal diseases, where the amount of CTs is considerably lower. Likely, this is related to the lack of standardization in terms of models, techniques, doses, optimal time points for evaluation and outcome measures of efficacy definition. The lack of similarity of the preclinical and the clinical settings, immunosuppressive treatments, or animal models that do not fully reflect human disease could also contribute to that discrepancy. Also noteworthy is that ophthalmic diseases are multifactorial and progress over time, so more longitudinal or chronic preclinical studies are needed, exploring diverse analyses such as visual tests, functional assessment, and cellular fate in the tissue, to support their use in patients. Other of the greatest advantages of preclinical studies is to be able to document the consequences of an intervention, both *in vivo* and *ex vivo*. In these types of therapies, it is very important to determine cell fate and histological changes that may occur, and that is only possible in the preclinical setting.

Furthermore, based on the data extracted from the included studies, it is difficult to extract a unique working mechanism to explain PnD advantages in the ophthalmology field. For some authors, the primary molecular mechanism involved is immunomodulation by the production of cytokines such as TGF-β or IL-10 (Barequet et al., 2008; Guo et al., 2011), the upregulation of essential survival pathways such as NF-κB (Park et al., 2018), and modifying cellular adhesion and motility (Lee et al., 2004). However, each PnD has different properties and that needs to be addressed.

In order to gather all available data on PnD, the SPRINT consortium established a publication database on the Mendeley Reference Manager platform (https://www.mendeley.com/) and collected outcome data from selected publications in an Access database (Microsoft, Redmont WA, United States of America), linking PnD types, dosage, route and time point of applications to functional effects. However, both databases remain to be further implemented in the scientific community (e.g. by the International Placenta Stem Cell Society, IPLASS).

Recently, also new non-cellular therapies have emerged to replace certain limitations of the use of live cells, such as maintaining viability upon allogeneic administration. In particular, extracellular vesicles derived from mesenchymal stromal cells are showing promising results (Thomi et al., 2019). However, it is difficult to draw conclusions about a common beneficial dose to treat a disease. For that, advancing our knowledge of cell-based regenerative mechanisms is still critical to reaching success in eye diseases.

## 5 Conclusion

The eye is a good candidate for PnD-based therapies and ocular diseases have gained attention from both pre-clinical and clinical researchers. However, the number of studies is considerably low, a sign that much work is still to be done.

Selected pre-clinical studies and clinical trials converge in a number of features. They targeted the same ocular structures (cornea/ocular surface and retina/optic nerve). They tended to choose the same type of PnD according to the target tissue and also their route of administration. In the cornea/ocular surface, hAM was preferred and usually applied directly covering the ocular surface. In neuroretinal disorders, cells of umbilical or placental origin were injected in the eye.

In general, basic research reported favourable outcomes in all animal models of disease treated with PnD. From the clinical perspective, hAM proved useful in the symptomatic relief of bullous keratopathy, treating dry eye disease and in preventing glaucoma drainage device tube exposure. hAM was well tolerated and safe in treating corneal ulcers and epithelial defects; however, it did not seem to outperform bandage contact lenses. In pterygia surgery and in scleral thinning, the use of isolated hAM should be discouraged. Regarding neuroretinal diseases, the use of Wharton’s Jelly MSC seems very promising, giving hope to otherwise irreversible diseases.

Pre-clinical studies are very important because they ought to provide necessary evidence supporting translation to the clinic. This includes efficacy and safety data, as well as insights into mechanisms of action. What stands out from our selection of pre-clinical studies is a lack of standardization among them. Also, until now, they have failed to provide an explanation on how exactly do these PnD work and their fate.

Clinical trials register in dedicated platforms is a valuable source of information. Surprisingly, the effort to register clinical trials is not accompanied by the effort to keep theirs records updated. Only a minority of CT provided results, despite displaying “complete status” and there are some whose status is “unknown”. Also, in the case of “withdrawn” CT, providing an explanation for this fact should be encouraged. Negative results are as important as positive results and becoming aware of them contributes to evolving knowledge.

In conclusion, PnD-based therapies seem to be beneficial in the treatment of several ocular diseases. However, much needs to be done both in the pre-clinical and in the clinical setting before they can be included in the daily ophthalmic practice.
