# Supplementary figures and images for: Insights and future directions for the application of perinatal derivatives in eye diseases: A critical review of preclinical and clinical studies
Source: Front Bioeng Biotechnol. 2022 Nov 8;10:969927. doi: 10.3389/fbioe.2022.969927 (PMC9679153; doi:10.3389/fbioe.2022.969927)

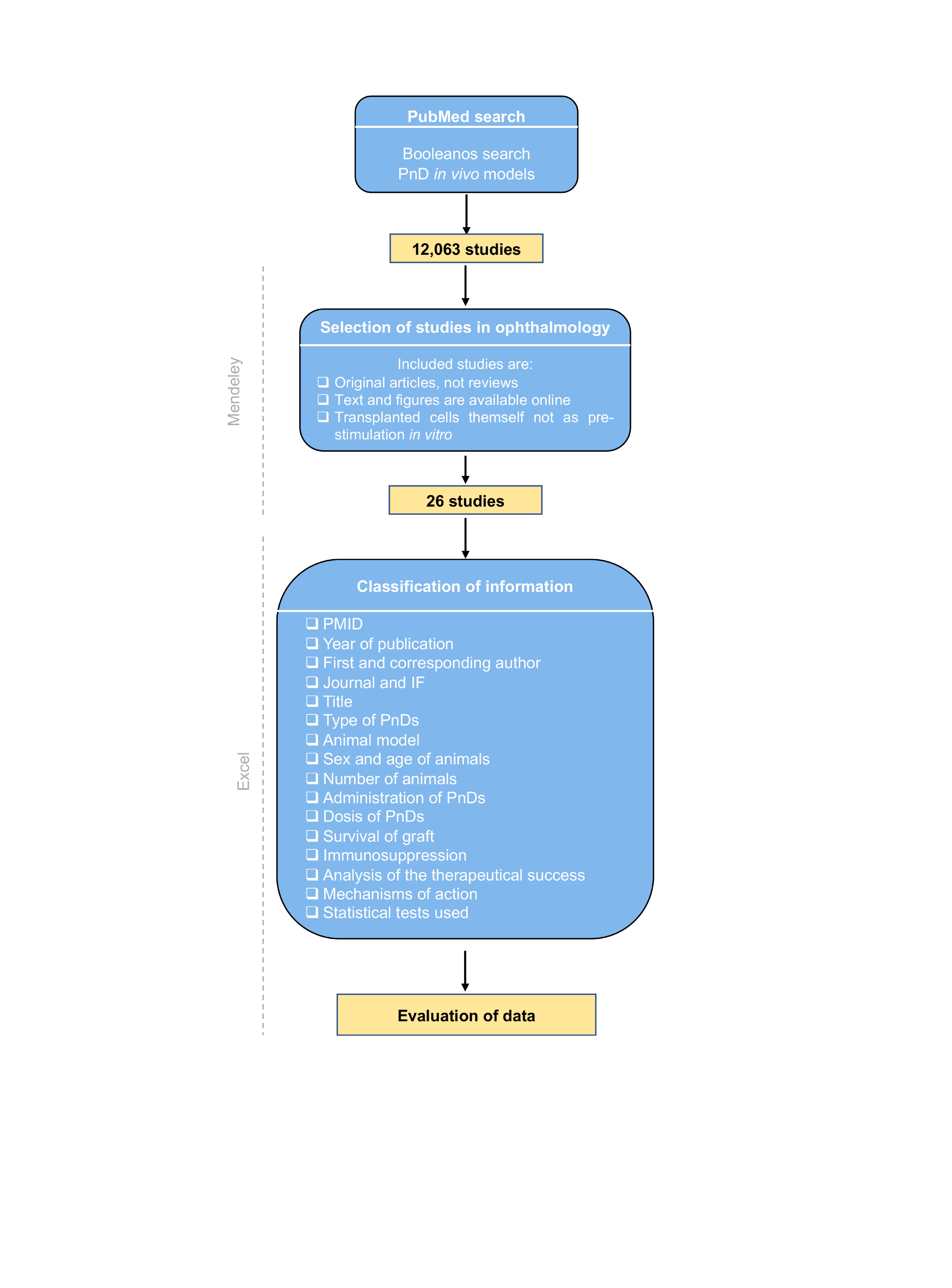

Supplement: Supplementary file 1 [file Image1.jpg]
